# A novel Fanconi anaemia subtype associated with a dominant-negative mutation in *RAD51*

**DOI:** 10.1038/ncomms9829

**Published:** 2015-12-18

**Authors:** Najim Ameziane, Patrick May, Anneke Haitjema, Henri J. van de Vrugt, Sari E. van Rossum-Fikkert, Dejan Ristic, Gareth J. Williams, Jesper Balk, Davy Rockx, Hong Li, Martin A. Rooimans, Anneke B. Oostra, Eunike Velleuer, Ralf Dietrich, Onno B. Bleijerveld, A. F. Maarten Altelaar, Hanne Meijers-Heijboer, Hans Joenje, Gustavo Glusman, Jared Roach, Leroy Hood, David Galas, Claire Wyman, Rudi Balling, Johan den Dunnen, Johan P. de Winter, Roland Kanaar, Richard Gelinas, Josephine C. Dorsman

**Affiliations:** 1 Department of Clinical Genetics, VU University Medical Center, Van der Boechorststraat 7, Amsterdam 1081 BT, The Netherlands; 2 Luxembourg Centre for Systems Biomedicine, House of Biomedicine, 7 Avenue des Hauts-Fourneaux, Esch/Alzette L-4362, Luxembourg; 3 Institute for Systems Biology, 401 Terry Avenue North, Seattle, Washington 98109-5234, USA; 4 Division of Biological Stress Response, The Netherlands Cancer Institute, Plesmanlaan 121, Amsterdam 1066 CX, The Netherlands; 5 Department of Genetics, Cancer Genomics Center, PO Box 2040, Rotterdam 3000 CA, The Netherlands; 6 Department of Radiation Oncology, Erasmus Medical Center, PO Box 2040, Rotterdam 3000 CA, The Netherlands; 7 Lawrence Berkeley National Laboratory, 1 Cyclotron Road, Berkeley, California 94720, USA; 8 Department of Paediatric Oncology, Hematology and Clinical Immunology, Center for Child and Adolescent Health, Medical Faculty, Heinrich Heine University, Moorenstrasse 5, 40225 Düsseldorf, Germany; 9 Deutsche Fanconi-Anämie-Hilfe e.V., Böckenweg 4, 59427 Unna, Germany; 10 Mass Spectrometry and Proteomics Facility, The Netherlands Cancer Institute, Plesmanlaan 121, Amsterdam 1066 CX, The Netherlands; 11 Pacific Northwest Diabetes Research Institute, 720 Broadway, Seattle, Washington 98122, USA; 12 Department of Human and Clinical Genetics, Leiden University Medical Center, Albinusdreef 2, Leiden 2333ZA, The Netherlands

## Abstract

Fanconi anaemia (FA) is a hereditary disease featuring hypersensitivity to DNA cross-linker-induced chromosomal instability in association with developmental abnormalities, bone marrow failure and a strong predisposition to cancer. A total of 17 FA disease genes have been reported, all of which act in a recessive mode of inheritance. Here we report on a *de novo* g.41022153G>A; p.Ala293Thr (NM_002875) missense mutation in one allele of the homologous recombination DNA repair gene *RAD51* in an FA-like patient. This heterozygous mutation causes a novel FA subtype, ‘FA-R', which appears to be the first subtype of FA caused by a dominant-negative mutation. The patient, who features microcephaly and mental retardation, has reached adulthood without the typical bone marrow failure and paediatric cancers. Together with the recent reports on RAD51-associated congenital mirror movement disorders, our results point to an important role for RAD51-mediated homologous recombination in neurodevelopment, in addition to DNA repair and cancer susceptibility.

## Abstract (web summary)

Fanconi anaemia is an inherited disorder characterised by developmental abnormalities, bone marrow failure and predisposition to cancer. Here the authors report a *de novo* mutation in the DNA repair gene *Rad51* in an atypical subtype of Fanconi anaemia.

## Graphical abstract

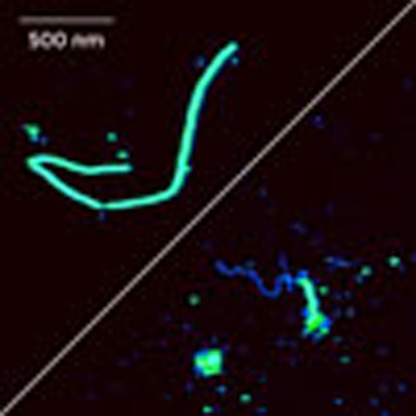

Fanconi anaemia (FA) is a rare recessive genetically heterogeneous chromosomal instability disorder with both autosomal and X-linked inheritance. All reported cases so far displayed a recessive mode of inheritance. FA is characterized by multiple congenital abnormalities, haematological defects and a high predisposition to a diversity of cancers. including childhood acute myeloid leukaemia and head and neck cancers. Patient-derived cells are extremely sensitive to DNA interstrand cross-linking agents, such as mitomycin C (MMC) or diepoxybutane (DEB)^1^. Standard FA diagnosis therefore involves the determination of chromosomal breaks and radial formation after exposure to DEB or MMC.

FA is a well-known example of how the study of a rare genetic disease can reveal novel molecular pathways. FA proteins were found to act together in a distinct genome maintenance pathway referred to as the FA/BRCA pathway, which is involved in the repair of DNA–DNA cross-links as well as DNA double-strand breaks^2^,^3^. Central in the pathway is the monoubiquitination of the FA proteins FANCD2 and FANCI, which is carried out by a multi-protein complex consisting of FANCA, -B, -C, -E, -F, -G, -L and -M. The FA proteins FANCD1 (BRCA2), -J (BRIP1), -N (PALB2), -O (RAD51C), -P (SLX4) and -Q (XPF) all act downstream of this monoubiquitination step. Recently, also biallelic *BRCA1* mutations have been described to cause a FA-like syndrome (‘FA-S')^4^.

Loss of the DNA repair function is thought to underlie the cancer predisposition. The FA/BRCA pathway research focused mainly on the different steps in DNA repair through homologous recombination (HR). This pathway is essential for accurate repair of double-strand breaks in mammalian cells. The key step in HR is homologous strand exchange mediated by the RAD51 protein. RAD51 binds to single-stranded DNA (ssDNA) in an adenosine triphosphate (ATP)-dependent manner to form a nucleoprotein filament able to invade duplex DNA and search for homology; RAD51 directly interacts with the FA protein BRCA2. However, RAD51 has not been identified as a FA protein so far. Furthermore, despite the extensive work to elucidate the function of FA proteins in response to DNA interstrand cross-link damage, the mechanism by which the FA/BRCA pathway responds to this genotoxic insult is not fully understood.

In this study, we describe an atypical adult male FA patient who had been excluded from all known FA subtypes by standard FA diagnostic DNA mutation analysis. This patient does not display the typical FA-related bone marrow failure and cancer, but features mental retardation. We sequenced the whole genomes of the patient, a healthy sister and the parents, and identified a *de novo* mutation in the *RAD51* gene of the patient as the disease-causing mutation. Proteomics experiments indicate that both the mutant and wild-type RAD51 are expressed in the patient-derived cells, suggesting a dominant-negative mode of action. In line with this, mutant RAD51 acts as a dominant negative in an inducible cell model. *In vitro*, the mutation affected crucial steps in HR: formation of a joint molecule between a processed DNA break and the repair template, ATP hydrolysis, DNA binding and filament formation. Moreover, mutant RAD51 can interfere with binding of wild-type RAD51 to DNA. Altogether, the data indicate that the heterozygous *RAD51* mutation causes a novel FA subtype, ‘FA-R', which appears to be the first subtype of FA caused by a dominant-negative mutation.

## Results

### Clinical and cellular phenotype of the FA-like patient

**Figure 1:**
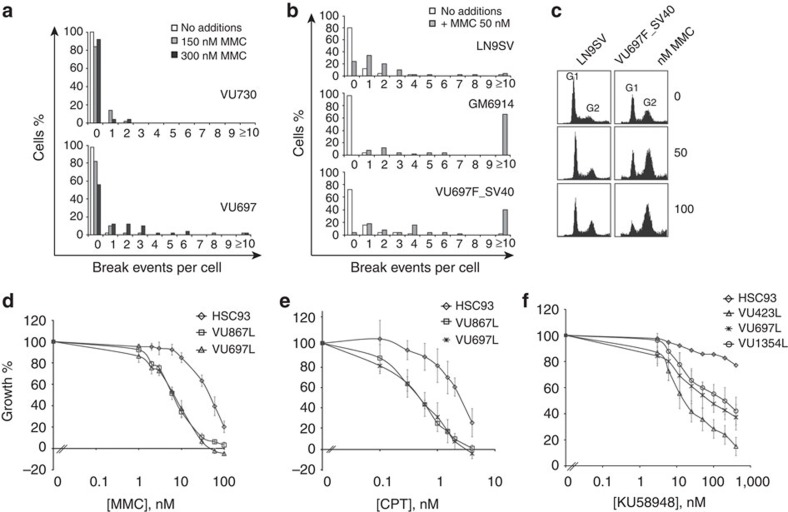
FA-characteristic cellular phenotypes of cells from the proband. (**a**) Number of cells (stimulated primary lymphocytes) with chromosomal breaks after addition of 150 and 300 nM MMC, from the proband (VU697) and his healthy sister (VU730); *P* value=0.0001 (*χ*^2^-test). (**b**) Spontaneous and MMC-induced chromosomal breakage in SV40-transformed fibroblasts from a healthy individual (LN9SV), a FANCA-deficient individual (GM6914), and the proband (VU697F_SV40); *P* value=0.005 (*χ*^2^-test). (**c**) Cell cycle distribution of SV40-transformed fibroblasts from a healthy individual (LN9SV) and from the proband without and with treatment of 50 or 100 nM MMC for 72 h. (**d**–**f**) Growth inhibition analysis of the proband's lymphoblast cells after treatment with DNA-damaging agents. Lymphoblast cell lines were assessed for sensitivity to MMC, camptothecin and PARP inhibitor, KU58948. VU697L is the proband's lymphoblast cell line, HSC93 is the wild-type control, VU867L is FANCM-deficient, VU423L is BRCA2-deficient and VU1354L is SLX4-deficient. Error bars indicate the s.d. from triplicate experiments.

The FA-like individual presented at 2.5 years of age with growth retardation, microcephaly, hydrocephalus, skeletal (thumb and radius) abnormalities, imperforate anus and an improperly formed left testicle. A positive DNA cross-linker (DEB)-induced chromosomal breakage test confirmed the suspicion of FA. The patient has now reached adulthood (23 years) lacking the typical FA features such as bone marrow failure and malignancies^5^. Somewhat atypical for FA was his strong learning disability associated with an intelligence quotient test score of about 70. Cellular tests using blood lymphocytes and SV40-transformed fibroblast cell lines established at age 5 years, confirmed the hypersensitivity to DNA cross-linkers (Fig. 1a,b). Following treatment with MMC, immortalized fibroblasts from the patient showed an excessive accumulation in the late S–G2 phase of the cell cycle (Fig. 1c). Using growth inhibition as an end point, patient-derived cell lines were hypersensitive not only to MMC, but also to camptothecin (CPT) and the poly-(adenosine diphosphate (ADP)-ribose) polymerase (PARP) inhibitor, KU58948 (Fig. 1d–f), suggesting that the cells are impaired in DNA repair. The results are consistent with a defect in the downstream branch of the FA/BRCA pathway, as western blot analysis demonstrated a normal level of FANCD2 monoubiquitination in the individual's lymphoblastoid and fibroblastoid cell lines (Supplementary Fig. 1).

### Whole-genome and -exome sequencing

**Figure 2:**
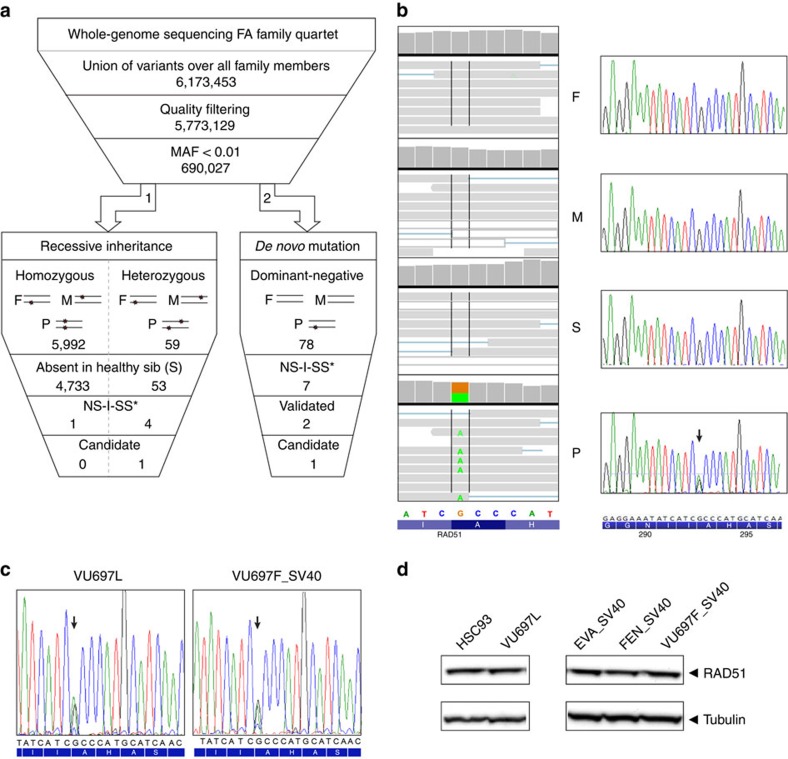
Genetic analysis of the proband and his family members. (**a**) Schematic overview of genomic analysis. Two types of analyses were performed. (I) Recessive mode of inheritance (expected for FA, arrow 1) and (II) dominant mode of inheritance; *de novo* mutation (arrow 2). NS-I-SS: non-synonymous, indel, splice-site variations. (**b**) Whole-genome sequence (WGS) data for the affected portion of the *RAD51* exon 10 visualized in integrative genomic viewer (IGV) demonstrating a heterozygous variant (variation indicated in green) for the proband, whereas the homozygous wild-type sequence was observed in all other family members (left panel). Sanger sequencing results for the same region and individuals are depicted in the right panel (variant indicated with arrow). (**c**) Sanger sequence data of complementary DNA from proband's lymphoblasts (VU697L; left) and fibroblasts (VU697F_SV40; right) showing the same variant (indicated with an arrow). (**d**) Western blot analysis of whole-cell extracts of wild-type (HSC93) and proband's (VU697L) lymphoblast cell lines and wild-type (EVA_SV40, FEN_SV40) and proband's (VU697F_SV40) immortalized fibroblast cell lines. RAD51 levels were determined with tubulin serving as a loading control.

To identify the disease-causing mutation(s), we performed whole-genome sequencing (WGS)^6^ on DNA from the proband, his parents and his unaffected sister. Relationship estimation^7^ based on the parents' genomes confirmed paternity and excluded consanguinity. Furthermore, we sequenced the exome from the patient to confirm variants identified by WGS. As recessive inheritance is typical for FA, we explored this possibility by examining genes harbouring either homozygous or compound heterozygous potentially disease-causing mutations (Fig. 2a; arrow 1). The candidate mutations had the following characteristics: very rare (minor allele frequency <0.01) and scoring positive in at least two out of four pathogenicity-prediction programs. Using data from the healthy sister as a negative control, this filtering approach resulted in only one candidate gene: *PRR12*. We tested this candidate gene with a prioritization approach tailored for the FA/BRCA pathway^8^. This tool considers the entire proteome exploiting the basic FA protein properties, protein–protein interactions (Molecular Interactions workflow of EnVision 2), protein function prediction (FuncNet) and literature mining (Nermal). With this analysis, *PRR12* scored only 1 point, while proteins from the top 150 had scores ranging from 8.500 (position 1) to 6.224 (position 150). In keeping with this, short interfering RNA knockdown experiments did not result in an FA-like phenotype^8^. Altogether, the causal genetic defect underlying the FA-like phenotype in the current patient does not seem to be associated with a recessive mode of inheritance.

### *De novo* mutation in *RAD51*

Therefore, we tested the hypothesis that a *de novo* mutation in one of the alleles might cause the disease through a dominant-negative mechanism (Fig. 2a; arrow 2). Seven variants in five genes were detected of which two (one in *RAD51* and the other in *DCHS2*) were validated by Sanger sequencing and predicted as possibly damaging. We then considered three factors for prioritizing these two genes: (i) an apparently relevant gene function, such as DNA interaction/repair, (ii) protein interactions with known FA proteins and (iii) FA protein-like properties. Considering these criteria, *RAD51* turned out to be the prime candidate since RAD51 mediates DNA strand exchange, the key step in HR repair^9^, binds the FA proteins BRCA2 and PALB2 (ref. 10) and has a high score (ranking 65, scoring 6.377) with the FA-ranking tool^8^. *RAD51* is a member of the *RAD51* family of related genes, of which *RAD51C* had already been recognized as an FA gene^11^. We furthermore confirmed the *RAD51* variant as the top candidate with two independent variant filtering workflows: ingenuity variant analysis and the family genomics workflow^12^.

### Stable expression of mutant RAD51 protein

The identified *RAD51* mutation, g.41022153G>A (hg19, chr15; Fig. 2b, left panel), is a novel mutation, not previously observed in human DNA, as determined by interrogating the publicly accessible variant databases. The mutation affects an amino acid (aa) at a highly conserved position, and results in a substitution, p.Ala293Thr (NM_002875), that is predicted to be damaging by three different tools (see Supplementary Methods). The variant was also found in the exome data. Subsequent Sanger sequencing was consistent with the WGS and exome data for the patient and family members (Fig. 2b, right panel). Furthermore, the mutation was also identified in complementary DNA (c.877G>A) obtained from proband-derived lymphoblasts and from skin fibroblasts (Fig. 2c). The latter finding excludes the possibility that the mutation was acquired during lymphoblast cell culturing and demonstrates that the mutant allele could be stably expressed at the messenger RNA level. Subsequent interrogation of RAD51 protein expression by western blot analysis indicated that patient-derived cells expressed similar levels of total RAD51 as control cells, suggesting that both wild-type and mutant alleles are indeed expressed (Fig. 2d; Supplementary Fig. 2). Furthermore, with mass spectrometry we confirmed the presence of RAD51^A293T^ mutant at the protein level in the patient-derived fibroblast cell line (Supplementary Fig. 3).

### Mutant RAD51 sensitizes to DNA-damaging agents

**Figure 3:**
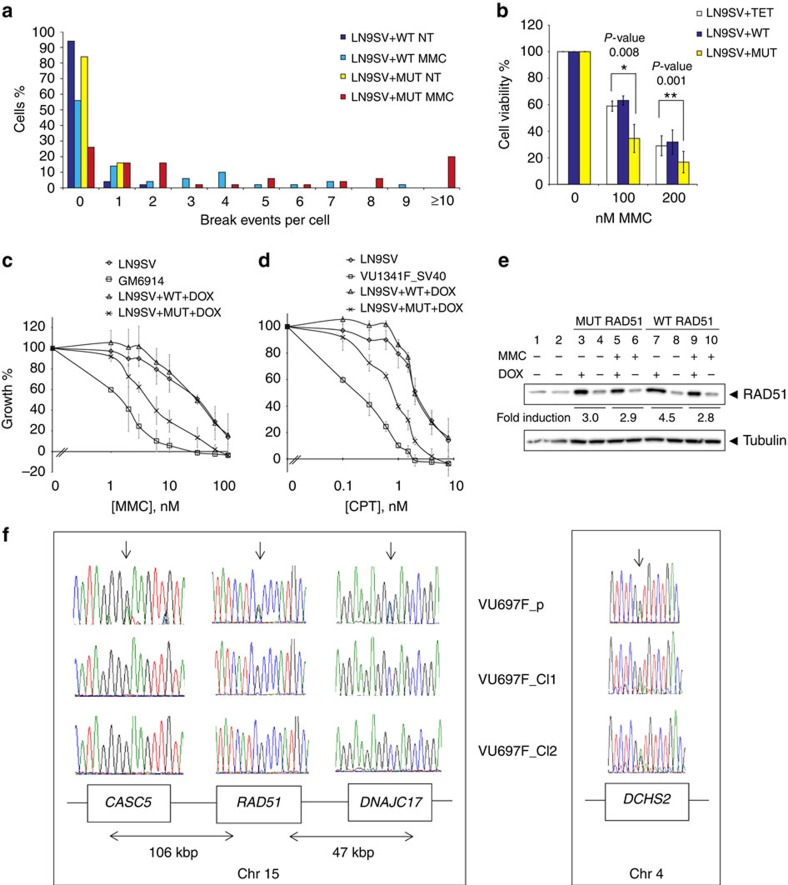
Functional assessment of the RAD51 mutant. (**a**) Analysis of inducible cell model. Chromosomal breaks. An increased number of cells with chromosomal breakage after treatment with MMC was observed for cells in which the expression of the mutant RAD51 allele (LN9SV+MUT) was induced compared with cells in which the wild-type RAD51 allele (LN9SV+WT) was induced; *P* value=0.001 (*χ*^2^-test). NT, ‘no treatment'; MMC, ‘treatment for 48 h with 50 nM MMC'. (**b**) CellTiter-Blue test. The viability of cells expressing the mutant allele (LN9SV+MUT) was compromised after treatment with MMC when compared with the cells expressing the wild-type allele (LN9SV+WT) or the wild-type cells transduced with the empty vector (LN9SV+TET). The average of three experiments is shown. (**c**) Growth inhibition test (cell counting). Wild-type cells expressing the mutant RAD51 allele are sensitive to MMC compared with wild-type cells expressing the wild-type RAD51 allele. Error bars indicate the s.d. from triplicate experiments. (**d**) Same test as in **c**, but with camptothecin (CPT). Both growth inhibition experiments were performed in triplicate. (**e**) Doxycyclin-inducible expression of mutant (lanes 3–6) and wild-type RAD51 (lanes 7–10) with or without treatment of MMC. Lanes 1 and 2 contain extracts from untransduced LN9SV wild-type cells. Levels of the RAD51 were determined with tubulin serving as control. (**f**) Analysis of revertant clones. Sanger sequence data show loss of the mutant *RAD51* allele together with LOH of single-nucleotide polymorphisms in the neighbouring genes *CASC5* and *DNAJC17* (left panel). The *DCHS2* variant on chromosome 4 is retained in both revertant clones (right panel).

It is striking that exactly the same substitution of the yeast orthologous aa residue (p.Ala351Thr) of RAD51 has been demonstrated to yield a semi-dominant-negative phenotype^13^. We thus hypothesized that the human counterpart would have a similar effect. To test this, we generated a fibroblast cell line with inducible expression of the human mutant *RAD51*, and a cell line with the wild-type gene as control. Expression of the RAD51 mutant gave rise to elevated spontaneous and MMC-induced chromosomal breaks, when compared with the same cell line that overexpressed the wild-type protein (Fig. 3a). Furthermore, the induced expression of the variant allele sensitized the cells to killing by the cross-linking agents MMC (Fig. 3b,c) and CPT (Fig. 3d). Although total levels of RAD51 are clearly higher in the induced cell lines, expression of the induced wild-type and mutant RAD51 proteins was in the same range, excluding the possibility that the observed negative effects on cell growth could be attributed to higher expression levels of the mutant protein (Fig. 3e; Supplementary Fig. 4)^14^. Furthermore, the data support the notion that the mutant protein is not destabilized by the mutation (see Fig. 2d).

### Spontaneous reversion by LOH

To provide additional evidence for the disease causality of the *RAD51* mutation, we generated spontaneous MMC-resistant revertants of the patient-derived fibroblastoid cell line via expanding the cells at a relatively mild selective pressure of 12.5 nM MMC. Of 58 isolated clones, in 7 (12%) only the wild-type *RAD51* allele could be detected via DNA sequencing. To discriminate between loss of heterozygosity (LOH) and back mutation as the underlying mechanism, we also sequenced the neighbouring genes of *RAD51*, *CASC5* and *DNAJ17.* The data indicated that LOH had occurred with retention of the wild-type *RAD51* allele (Fig. 3f, left panel). Importantly, no clones were observed with only the mutant allele. The observed direction of LOH under selective pressure reinforces the notion that the *RAD51* mutation is indeed causal for the FA-like cellular features. Moreover, the other potential deleterious mutation, at the *DCHS2* locus, was retained in the revertant clones, excluding this candidate gene as causal for the FA cellular phenotype (Fig. 3f, right panel). Thus based on the combined data, the RAD51 defect is underlying the DNA repair phenotype.

### Subcellular localization of RAD51 not affected

To determine whether the observed defect in DNA repair could be caused by mislocalization of RAD51 in the mutant cells, we performed subcellular fractionation experiments. We did not observe significant differences in RAD51 subcellular localization between wild-type and patient cells (Supplementary Fig. 5). Furthermore, we were able to detect RAD51 foci upon challenge with DNA-damage-inducing agents MMC and hydroxyurea (Supplementary Fig. 6). Altogether, this suggests that in the presence of both the wild-type and the mutant allele, some residual activity of RAD51 is retained. This is in line with the observed embryonic lethality associated with *Rad51* knockout in mice^15^.

### RAD51^A293T^ impairs D-loop formation

**Figure 4:**
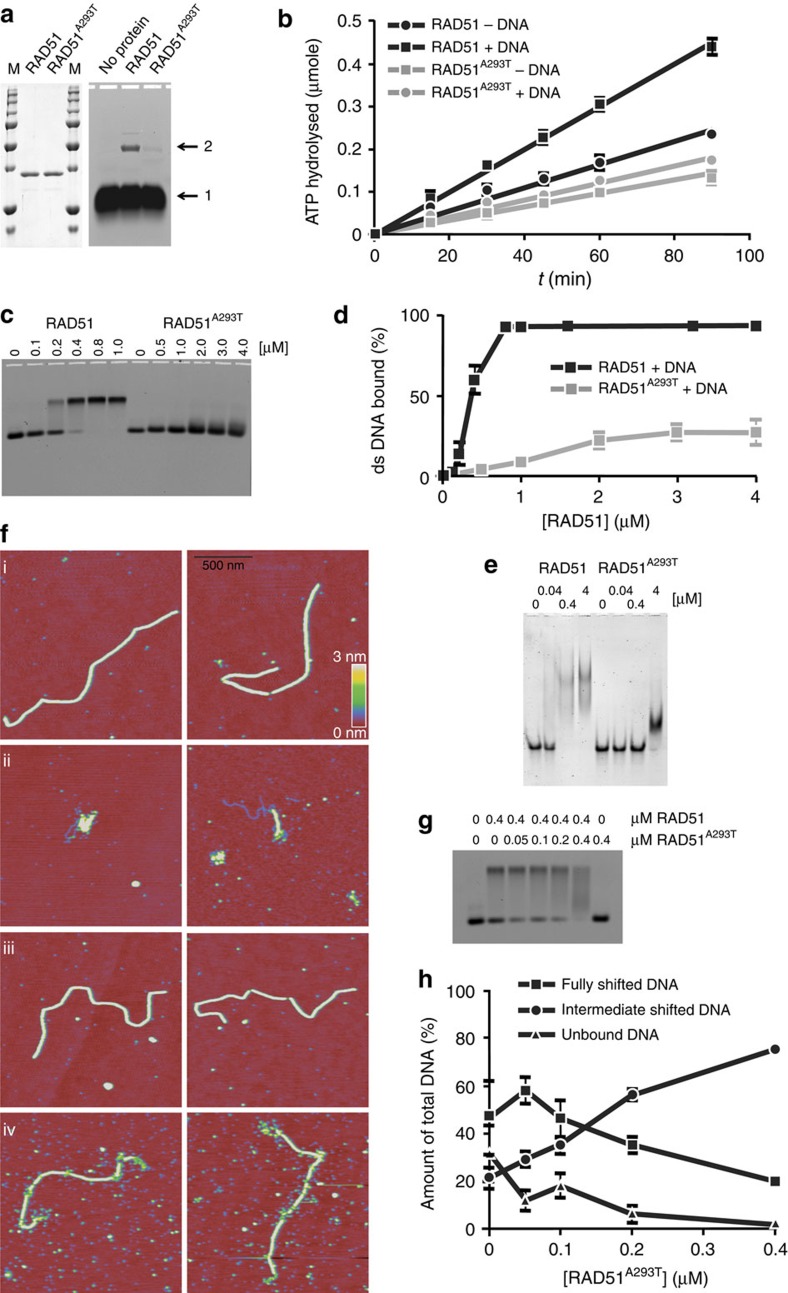
Biochemical studies of RAD51. (**a**, left panel) RAD51 purification. Wild-type RAD51 and RAD51^A293T^ were purified and 1 μg of protein was electrophoresed through an SDS-containing polyacrylamide gel; M, molecular weight marker. (**a**, right panel) D-loop assay. The efficiency of D-loop formation, reporting on joint molecule formation and DNA strand-exchange activity, was tested; arrow 1 indicates the free oligonucleotide, arrow 2 indicates the strand-exchange product between the oligonucleotide and a homologous supercoiled plasmid. (**b**) ATPase assay. The ATPase activity of wild type and RAD51^A293T^, either in the absence or presence of single-stranded DNA, was measured using thin-layer chromatography. The amount of ATP hydrolysed is plotted as a function of time. Error bars represent the s.e.m. from three experiments. (**c**) EMSA dsDNA. Fluorescently labelled double-stranded DNA molecules (66 bp) were incubated with the indicated concentrations of wild-type RAD51 or RAD51^A293T^ in the presence of ATP and Ca^2+^ and electrophoresed through a native agarose gel. (**d**) Quantification of the percent DNA bound as a function of protein concentration. Error bars indicate the s.e.m. of five to seven experiments. (**e**) EMSA ssDNA. Fluorescently labelled single-stranded DNA molecules (66 nt) were incubated with the indicated concentrations of wild-type RAD51 or RAD51^A293T^ in the presence of ATP and Ca^2+^ and electrophoresed through a native polyacrylamide gel. (**f**) Scanning force microscopy (SFM). SFM images of filaments formed by wild-type human RAD51 on double-stranded DNA in the presence of 100 mM KCl (i) and mutant RAD51^A293T^ (ii). Examples of nucleoprotein filaments formation on double-stranded DNA in the presence of 30 mM KCl by wild-type (iii) and mutant (iv) RAD51. All images are 1.5 × 1.5 μm and height is represented by colour in the range of 0–3 nm, red to yellow as shown in the scale bar. (**g**) EMSA. Double-strand DNA binding was assessed by EMSA in reaction mixtures containing the indicated concentrations of wild-type and mutant RAD51. (**h**) Quantification of the percent of unbound DNA and protein–DNA complexes detected by EMSA as a function of the RAD51^A293T^ protein concentration.

To determine the molecular mechanism underlying the DNA repair defect observed in cells expressing RAD51^A293T^, we performed a number of biochemical experiments. Wild-type RAD51 and RAD51^A293T^ were produced in *Escherichia coli* and purified as described previously (Fig. 4a, left panel)^16^. Pivotal steps in HR, which are catalysed by RAD51, are recognition of homology and strand exchange between homologous DNA partners to form a joint molecule between a processed DNA break and the repair template. A biochemical assay reporting on these activities is D-loop formation, which measures homology-dependent joint molecule formation between a single-strand oligonucleotide and a supercoiled plasmid^17^. The p.Ala293Thr mutation in RAD51 reduced the formation of D-loop intermediates by ninefold (Fig. 4a, right panel). The RAD51^A293T^ protein is thus severely defective in joint molecule formation, a key intermediate step in DNA repair by HR.

### Impaired ATPase activity and DNA binding by RAD51^A293T^

To uncover the stage of HR that is negatively impacted by the p.Ala293Thr mutation, we tested several functions of RAD51 related to its role in HR. We tested whether the mutation affected the ATPase activity of RAD51. As previously reported, wild-type RAD51 displayed a low constitutive ATPase activity that is stimulated approximately twofold by ssDNA (Fig. 4b)^18^. In contrast, RAD51^A293T^ had a somewhat lower baseline ATPase activity that could not be stimulated by DNA (Fig. 4b). The lack of DNA-stimulated ATPase activity of the mutant protein suggests a lower affinity for binding to DNA. To directly test this possibility, we performed electrophoretic mobility shift assay (EMSA) experiments using both double-stranded and ssDNA. Double-strand DNA-binding affinity of wild-type RAD51 and RAD51^A293T^ was tested under identical conditions. DNA binding by wild-type RAD51 resulted in a discrete band in the EMSA (Fig. 4c). Quantification revealed that half of the DNA was bound at 0.3 μM and binding was saturated at 0.8 μM RAD51 (Fig. 4d). By contrast, RAD51^A293T^ did not produce a discrete band. Instead a slight smear was detected, just above the unbound double-stranded DNA (dsDNA), but only at protein concentrations an order of magnitude higher than needed for efficient DNA binding by wild-type RAD51 (Fig. 4c). When ssDNA was used, wild-type RAD51 binding was saturated at a concentration of 0.4 μM (Fig. 4e), while it took a 10-fold higher concentration for RAD51^A293T^ to shift all of the DNA probe. Furthermore, DNA binding by RAD51^A293T^ resulted in a qualitatively different protein–DNA complex with faster mobility than that formed between wild-type RAD51 and ssDNA (Fig. 4e). We conclude that the p.Ala293Thr mutation attenuates the DNA-stimulated ATPase activity of RAD51 and negatively affects the binding of RAD51 to both single- and dsDNA.

### Distorted RAD51^A293T^ nucleoprotein filament formation

A key structural intermediate in DNA repair through HR is the RAD51 nucleoprotein filament^19^. Although EMSA experiments allow quantification of protein–DNA complexes, they do not reveal any information on their architectural arrangement. By contrast, several structural features of RAD51–DNA complexes can be quantified by scanning force microscopy (SFM)^20^. Wild-type RAD51 was incubated with 3.9-kbp dsDNA in the presence of Ca^2+^ and ATP, and the reaction mixture was analysed by SFM. Under these conditions, RAD51 forms stable highly regular nucleoprotein filaments of defined length that completely cover the DNA^20^, (Fig. 4f, i). With the same conditions, some of the observed DNA did have bound RAD51^A293T^ protein (examples shown in Fig. 4f, ii), but the SFM analysis revealed that these DNA–RAD51 complexes differed markedly from the regular wild-type nucleoprotein filaments. DNA molecules were only partly covered with protein, in the form of short RAD51^A293T^ filament patches, which tended to associate with each other and naked DNA along the same molecule making them difficult to analyse further (Fig. 4f, ii).

To analyse the effect of the p.Ala293Thr mutation on RAD51 nucleoprotein filaments in more detail, we assembled RAD51–DNA complexes in conditions expected to stabilize otherwise unstable filaments^21^ at 30 mM KCl instead of the 100 mM used above. Under these conditions, the p.Ala293Thr protein was able to form more extensive nucleoprotein filaments from which several structural aspects could be reliably quantified (Fig. 4f, iv). Filaments formed with wild-type protein look much the same in the lower- and higher-salt conditions (Fig. 4f, compare i with iii). In contrast to wild-type RAD51, the RAD51^A293T^ nucleoprotein filaments contain apparent gaps in protein coverage and irregular protein arrangement especially evident at the ends (Fig. 4f, iv). The differences in architecture were quantified by measuring the length and the height of the filaments. Binding of RAD51 to dsDNA changes the helical pitch such that the DNA molecule extends to 1.5-fold its B-form length^22^,^23^. If filament formation is not complete, DNA will be less extended and the filaments shorter. The wild-type RAD51 nucleoprotein filaments on the 3.9-kb DNA were 2,025±67 nm long, while filaments formed on the same-length DNA with RAD51^A293T^ were 1,752±105 nm long. Discontinuities in the filament are detected in SFM images as a reduction in height from the average of 2.2±0.4 nm to less than 1 nm. On this 3.9-kb DNA, we rarely observed discontinuities in filaments formed with wild-type RAD51; on average 0.29 locations with a height below 1 nm per filament (*n*=17 filaments). In contrast, discontinuities were frequent if filaments were formed with the RAD51^A293T^ protein; on average 17.4 locations below 1 nm observed per filament (*n*=25 filaments). The appearance of the filaments in SFM, their decreased length and more frequent discontinuities, indicate that although RAD51^A293T^ can form filaments on DNA, they are less stable and possibly defective specifically in filament elongation. Thus, the mutation interferes with the crucial function of RAD51 to form functional nucleoprotein filaments.

### RAD51^A293T^ interferes with wild-type RAD51 DNA binding

As RAD51 functions in the context of a helical nucleoprotein filament that requires repeated monomer–monomer contacts^22^, it is possible that defective DNA binding of the mutant could impose a dominant-negative effect on the function of wild-type RAD51. Indeed, titrating in RAD51^A293T^ in reaction mixtures containing dsDNA and a fixed amount of wild-type RAD51 affected the ratio of different protein–DNA complexes detected in the EMSA (Fig. 4g). At a concentration of 0.4 μM wild-type RAD51, both protein–DNA complexes and unbound DNA were detected (Fig. 4g). At a ratio of 1:8 mutant to wild-type protein, overall DNA binding increased slightly (Fig. 4h). This observation is consistent with RAD51^A293T^ being incorporated in the nucleoprotein filament formed by wild-type RAD51, as it does not bind DNA by itself at that concentration (Fig. 4c). At a 1:4 ratio, the relative amount of the fully shifted discrete band started to decrease, while protein–DNA complexes causing an intermediate shift were increased. At a 1:1 ratio, which still represents a protein concentration (0.4 μM) at which the RAD51^A293T^ protein by itself did not bind DNA, most of the shifted DNA detected had an intermediate mobility. Interestingly, under those conditions all unbound DNA had disappeared. Given that at a concentration of 0.8 μM wild-type RAD51 all DNA is present in a fully shifted discrete band, the disappearance of the fully shifted discrete band and the unbound DNA with the concomitant appearance of DNA with intermediate mobility suggests that mixed complexes containing both wild-type and mutant RAD51 form on DNA and these protein–DNA complexes are less stable than those containing only wild-type RAD51. We conclude that the presence of the RAD51^A293T^ protein quantitatively and qualitatively affects DNA binding by wild-type RAD51 supporting a dominant-negative effect of the mutant protein on the wild-type function.

**Figure 5:**
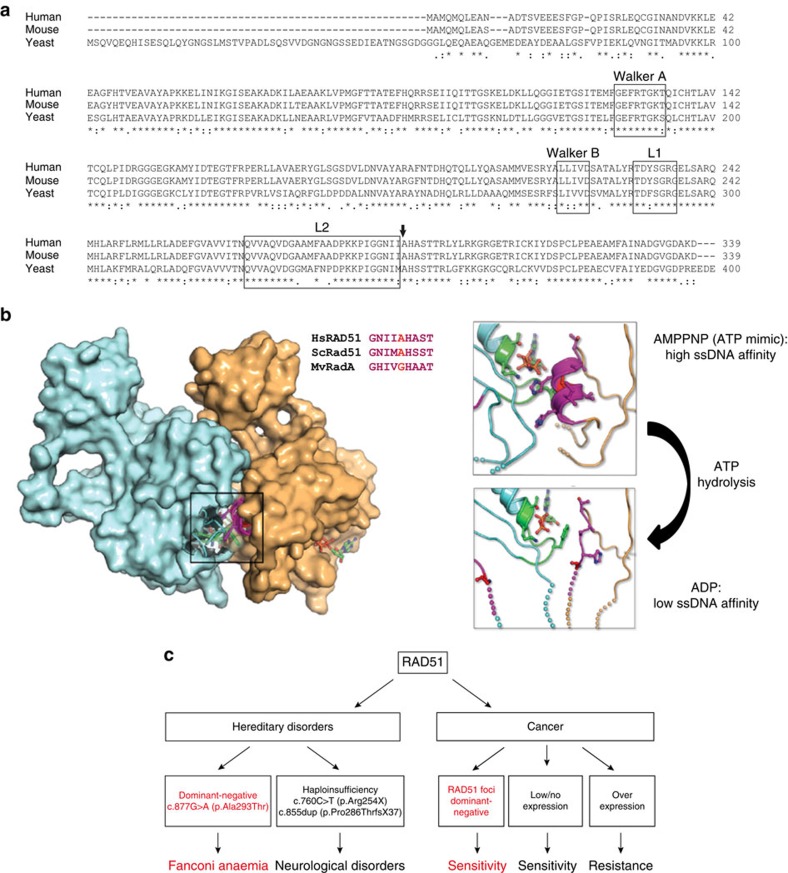
RAD51 in relation to human disorders. (**a**) ClustalW alignment of human, mouse and yeast RAD51 proteins demonstrating the conservation of the mutated aa (arrow). In addition the L1 and L2 important for DNA binding are shown, as well as the Walker A and Walker B regions, which have been implicated in nucleotide binding; the mutation is directly next to the L2 loop. (**b**) Crystal structures of *Methanococcus voltae* RADA (MvRadA, archaeal RAD51 homologue) have been solved in complex with the ATP mimic AMPPNP^52^ (PDB 2F1H) and with ADP^53^ (PDB 3FYH), providing the greatest structural insight into functionally relevant RAD51 forms. The AMPPNP—a non-hydrolysable ATP analogue—bound RAD51 dimer is shown in surface representation with each RAD51 monomer coloured differently. This shows that the motif surrounding the proband's mutation (magenta) is at the dimer interface (top). The equivalent residue to Ala293 in MvRadA is a glycine (red), although the surrounding motif is highly conserved (inset sequence alignment). Close-up views of the nucleotide-bound states of MvRadA (right) show that when bound to AMPPNP—a high-affinity ssDNA-binding state—the motif that contains the proband's mutation forms a small alpha-helix. This helix interacts with the bound nucleotide and the Walker A box (green). The region also neighbours the L2 loops that bind to ssDNA (spheres at the end of the L2 loops show regions of disorder in the crystal). In contrast, when bound to ADP—a low ssDNA-binding state—the proband's mutation motif has shifted position away from the nucleotide and Walker A box, and transitioned from an alpha-helix to a partially disordered loop with additional disorder seen in the L2 loops compared with the AMPPNP state. The position of the mutation in the protein supports the notion that mutation affects RAD51 protein dynamics. (**c**) Cartoon showing the different effects of changes of RAD51 on clinical phenotypes. A dominant-negative mutation can give rise to an FA-like disorder, haploinsufficiency (that is, lower levels) of RAD51 can give rise to a neurological disorder, while aberrant RAD51 expression levels in tumours have been associated with therapy response.

The RAD51-ATP-DNA filament catalyses the central DNA strand-exchange step of HR DNA repair by coordinated dynamic changes at multiple RAD51 interfaces (protein–protein, protein–ATP and protein–DNA; see also Fig. 5). In principle, even small changes at any of these dynamic interfaces could disturb function in a dominant manner. Our genetic and biochemical characterization now demonstrates that such mutations do exist in humans.

### RAD51 protein and human diseases

The p.Ala293Thr mutation is located in a highly conserved region of RAD51, which is involved in RAD51 monomer–monomer interactions^24^,^25^ (Fig. 5). The mutated aa is directly adjacent to the highly conserved so-called L2 loop, which is involved in DNA binding^26^ (Fig. 5a). The protein–interaction interface displays different conformations associated with ATP- or ADP-binding status (Fig. 5b right panel), which gives rise to high-affinity or low-affinity ssDNA binding, respectively. The region harbouring the mutation is thus implicated in RAD51 protein dynamics. Our data point to a crucial aa in this region whose alteration results in disturbed dynamic interactions evident as less stable DNA binding, loss of DNA-binding coordinated ATPase and disrupted filament structure. In the presence of the wild-type protein, apparently some function is retained, which may explain the viability of the FA-like patient. This study thus links a *RAD51* mutation to a novel clinical phenotype, that is, an FA-like disorder, which implies that the gene name *FANCR* may be added as a synonym to the official gene symbol, *RAD51*. Different types of mutations in human *RAD51* are thus associated with different clinical phenotypes (see Fig. 5c). A dominant-negative mutation gives rise to an FA-like phenotype, whereas haploinsufficiency of *RAD51* has been associated with congenital mirror movement disorder^27^. Importantly, the FA-like patient has not displayed mirror movements. So far, no hereditary disorders have been linked to overexpression of RAD51.

Somatically acquired altered expression of RAD51 or absence of RAD51 foci in cancers has been linked to therapy response^14^,^28^. However, our data call attention to the potentially clinically relevant finding that cells displaying RAD51 foci may still harbor subtle dominant mutations in RAD51, and could be sensitive to drugs, such as PARP inhibitors. Importantly, our finding expands the scope of cancer patients who may benefit from treatment with PARP inhibitors.

## Discussion

Until now FA research has primarily focused on malignancies and bone marrow failure, which constitute the prime life-threatening symptoms. Notably, not all FA patients acquire bone marrow failure. Indeed, this phenotype has not been associated with patients affected by biallelic mutations in *BRCA2* (ref. 29) and *RAD51C*^11^ who, together with the proband of the current study, seem to compose a distinct subtype. Intriguingly also the recently described cases of biallelic carriers of *BRCA1* mutations^4^,^30^, had reached adulthood without bone marrow failure. Instead these two individuals developed breast and serous ovarian cancer as adults, respectively. It could be hypothesized that individuals with a *de novo RAD51* mutation also have an increased susceptibility for cancer as adults and thus careful surveillance is warranted.

In addition, our study highlights the association of mental retardation/aberrant neurological functioning as a clinical feature linked to RAD51. In early reviews, a link between FA and mental retardation/central nervous system has been established, albeit at a low frequency^31^,^32^,^33^. Also the two cases with biallelic *BRCA1* mutations were found to be associated with developmental delay, while for one of the patients, mental retardation was mentioned explicitly. Thus, it could be hypothesized that absence of paediatric bone marrow failure, but presence of mental retardation/brain disorders is preferentially associated with individuals with defects in proteins of the downstream branch of the FA pathway. Intriguingly, FA cells are also exquisitely sensitive towards acetaldehyde, a metabolite of ethanol linked to fetal alcohol syndrome, which is an important cause of learning disability^34^,^35^.

Thus our study seems to warrant further exploration on the role of the FA/BRCA pathway in the (developing) brain and how this pathway may counteract toxic insults, such as exogenous ethanol. In addition, our data emphasize that dominant mutations can be responsible for diseases usually thought of as (autosomal) recessive and that genome-wide sequence data of family members combined with mechanistic studies may be necessary to pinpoint the responsible gene.

## Methods

### Ethics statement

Informed consent was obtained from the family involved in the study. Approval for this research was obtained from the Institutional Review Board of the VU University Medical Center adhering to local ethical standards of the Netherlands. WGS protocols and data analysis were approved by the Western Institutional Review Board on behalf of the Institute for Systems Biology in the USA.

### DNA sequence analysis

Genomic DNA was isolated from lymphoblastoid cell lines of the patient, the healthy sibling and the parents. WGS was performed at Complete Genomics Inc.^6^ (CG) using their standard sequencing pipeline, version 2.0.2.26. For further analysis only small variants (single-nucleotide polymorphisms and small insertions, deletions and block substitutions) from the CG ‘var' file were considered, which reports variants compared with the reference genome (NCBI build 37.2) up to a length of about 50 nucleotides. CGAtools (CG Analysis Tools) version 1.5 was used as provided by CG (http://cgatools.sourceforge.net) for subsequent analysis. With an in-house WGS analysis pipeline, we searched for recessive, compound heterozygous and *de novo* variants (Fig. 2). First, we combined all variants from all genomes of every family member into the union of variants using the CGAtools listvariant command and CG ‘var' files as input. Then, we used the CGAtools testvariant command to test each genome for the presence of each allele at every variant position. We next applied the following filtering steps: (1) we removed variants that were not called in at least one genome as high-quality calls (VQHIGH) by CG. (2) Next, we filtered for rare variants having a minor allele frequency equal or smaller than 1% in the European American population of the 1000genomes project^36^ and the NHLBI ESP exomes (https://esp.gs.washington.edu), as well as in the control data set CG69 provided by CG. (3) Variants were filtered out, for which in the index patient both alleles were called reference. If one or both alleles of the index patient were not called, the variant was not filtered out at this stage. (4) When applying a recessive inheritance model, we filtered for variants that were either called homozygous in the index patient and not in the unaffected sibling and heterozygous in the parents or compound heterozygous allowing for no call alleles. Here we filtered for two different heterozygous variants within the same gene coming from different parental genomes and both not present in the unaffected sibling. (5) Variants were annotated using ANNOVAR^37^ using the NCBI RefSeq Release 60 and Ensembl Release 74 genome annotations. Only exonic variants were kept that were annotated as non-synonymous, frameshift insertion/deletion, non-frameshift insertion/deletion/substitution, stop gain, stop loss and variants disrupting splice sites up to two bases into intron or exon. The remaining variants were then assessed using SIFT^38^, Polyphen^39^, MutationTaster^40^ and CADD^41^ for pathogenicity. Ingenuity Variant Analysis (Qiagen) and the Family Genomics Workflow (familygenomics.systemsbiology.net/software) provided independent candidate lists, and were used for confirmation of the primary analysis. Whole-exome sequencing was performed after enrichment with SureSelect Human All Exon Kit (Agilent) targeting ∼38 Mb, and sequenced on one lane of an Illumina GAIIx instrument using a paired-end sequencing protocol. To detect and evaluate SNVs and small indels, reads were mapped to the GRCh37 reference genome (hg19) by the Burrows–Wheeler Aligner^42^. Subsequently, SNVs and small indels were called using SAMtools^43^ and VarScan^44^. The resulting list of variants was annotated with ANNOVAR. Finally, manual inspection of relevant variants was carried out by visualizing the mapped reads in the Integrative Genome Browser (IGV)^45^.

### Sanger sequencing

Mutation confirmation by Sanger sequencing was done by direct sequencing of PCR products generated by RAD51-specific primers, 5′-GAAGAGAGACATTAGTTATTCGTT-3′ (forward) and 5′-ACGAGAGCATGTTAATTAGAT-3′ (reverse). PCR fragments were treated with shrimp alkaline phosphatase and exonuclease I for 30 min at 37 °C followed by incubation at 80 °C for 15 min. Sequencing reactions were carried out using 10 pM of primer with the Big Dye Terminator Cycle Sequencing kit (Applied Biosystems). Samples were analysed with an ABI 3730 DNA analyzer (Applied Biosystems).

### Relationship detection

The parents' genomes were compared using *GRAB*^7^. GRAB predicted ‘no relationship', indicating that the parents are not likely to have a relationship closer than 9th degree.

### Cell cultures and transformations

All cells used were from the VUmc Department of Clinical Genetics cell repository; cells were regularly tested for mycoplasma. Epstein–Barr Virus-immortalized B-cell lines from blood samples, were propagated in Roswell park memorial medium (RPMI) supplemented with 10% FCS (Gibco). SV40-transformed fibroblasts from skin biopsies were grown in DMEM (Gibco) containing 10% FCS.

The Lenti-X Tet-On 3G-inducible expression system (Clontech Laboratories) was used to express wild-type, mutant RAD51 and empty-vector constructs in the wild-type SV40-immortalized fibroblasts LN9SV. The generation of the mutant RAD51 construct was realized in a two-step PCR. For the first step, two separate PCR reactions were performed; (1) the forward oligo containing a BamHI site, 5′-acctGGATCCGCCACCATGGCAATGCAGATGCAGCTTG-3′ (cRAD51_*Bam*H1_F), was combined with the reverse oligo, 5′-CAATCTGGTTGTTGATGCATGGGTGATGATATTTCCTCCAATAGGTT-3′ spanning the mutation; (2) the forward oligo 5′-AACCTATTGGAGGAAATATCATCACCCATGCATCAACAACCAGATTG-3′ spanning the mutation, was combined with the reverse oligo containing a MluI site, 5′-attcACGCGTTCAGTCTTTGGCATCTCCCACTC-3′ (cRAD51_*Mlu*I_R). Subsequently, the PCR products were pooled and used as template in a PCR reaction using the cRAD51_*Bam*H1_F and cRAD51_*Mlu*I_R primers. The wild-type construct was established by amplification of complementary DNA derived from LN9SV using the cRAD51_*Bam*H1_F and cRAD51_*Mlu*I_R primers. Finally, the DNA sequences of the constructs were checked via Sanger sequencing. Doxycyclin to a final concentration of 200 ng ml^−1^ was added to the culture medium every 48 h to induce the expression of the constructs.

### Chromosomal breakage analysis

The chromosomal breakage assay was performed on heparinized venous blood from patient and control, SV40-transformed fibroblast cell lines from patient and controls were cultured for 72 h without or with MMC (Kyowa Hakko Co. Tokyo, Japan). In flasks, cells were treated with 200 ng ml^−1^ demecolcin (Sigma) for 30 min, harvested and then treated with 0.075 M KCl for 20 min at room temperature and fixed with 75% methanol, 25% acetic acid. Subsequently, cells were dropped onto glass slides and stained with 5% Giemsa (Merck)^46^. Per culture, 100 metaphases were scored from at least two coded slides (blind).

### Growth inhibition analysis

Growth inhibition assays on lymphoblasts and fibroblasts were performed in tissue culture flasks (25 cm^2^)^47^, which were grown in increasing concentrations of MMC, CPT or PARP inhibitor (KU58948). After the untreated cells made at least three population doublings, the relative cell number of each concentration compared with untreated cells was determined using a Coulter counter. All experiments were performed at least three times.

### Cell cycle analysis

SV40-immortalized fibroblasts were cultured for 72 h with or without MMC (50 nM or 100 nM). Cells were harvested by trypsinization and permeabilized in buffer containing 100 mM Tris-HCL (pH 7.5), 150 mM NaCl, 0.5 mM MgCl2, 1 mM CaCl2, 0.2% bovine serum albumin (BSA) and 0.1% NP-40 followed by staining of DNA with PI/RNase staining buffer (BD Biosciences). The cell cycle distribution was subsequently analysed by flow cytometry using a Particle Analysing System (PAS, Partec). The experiment was performed in duplicate.

### Cell viability assay

Fibroblast cell lines were seeded at 1,000–2,000 cells per well in 96-well plates. After 4 h, MMC-containing medium was added. Cells were incubated for 5 days, and their survival rates were measured using a CellTiter-Blue kit (Promega) following the manufacturer's instructions on a Tristar LB 941 (Berthold Technologies). The experiment was performed in triplicate. To compare cell growth in the different groups, the *t*-test was used.

### Selection and assessment of revertant fibroblast clones

VU697F fibroblasts (2,000 cells) were seeded per 10-cm dish (Falcon) in DMEM (Gibco) supplemented with 10% FCS, penicillin (100 U ml^−1^) and streptomycin (100 μg ml^−1^). To obtain spontaneous revertant clones, 12.5 nM MMC was added to the culture dishes. Cells were allowed to proliferate for 14–21 days after which single-cell-derived-colonies were picked. The cells from the single colonies were expanded, DNA was isolated (QIAmp DNA Micro kit, Qiagen) and the *RAD51* mutation was analysed via PCR followed by Sanger sequencing (forward primer: 5′-CCACCGCCCTTTACAGAACA-3′; reverse primer: 5′-ACAGGGGAGAGGCATATCAAT-3′). To confirm LOH, single-nucleotide polymorphisms in the neighbouring genes of *RAD51*, *CASC5* and *DNAJC17*, were assessed by Sanger sequencing (*CASC5* forward primer: 5′-CAGTGATCTGGAAGTCACCGA-3′; *CASC5* reverse primer: 5′-CACTGGATCCACAAACAGCAG-3′; *DNAJC17* forward primer: 5′-GACCCCATACTTCTGTGCTGT-3′; *DNAJC17* reverse: 5′-TGTGGGTGGGACAAATCACC-3′).

### Protein analysis

For FANCD2 analysis, 200,000 cells were lysed using 50 mM Tris-HCl pH 7.4, 150 mM NaCl, 1% NP-40 supplemented with protease inhibitors (Complete mini protease inhibitors tablet; Roche) and phosphatase inhibitors (PhosStop inhibitor coctail tablets, Roche). Cells were incubated 10 min on ice, followed by centrifugation at 4 °C for 10 min in an eppendorf centrifude 5415R at 13,200 r.p.m. Whole-cell extracts for RAD51 protein levels were prepared in lysis buffer (50 mM Tris-HCL, pH 7.5, 250 mM NaCl, 5 mM EDTA, 0.1% Triton X-100, supplemented with Complete mini protease inhibitors tablet; Roche). Protein concentration was determined with the Bradford method. Cell fractionation was performed using the Subcellular Protein Fractionation Kit (Thermo Scientific) according to the manufacturer's instructions. Protein extracts/subcellular fractions were resolved on a Novex 8-16% Tris-glycine 1-mm minigel (Invitrogen) and subsequently transferred to polyvinylidene difluoride membrane (Immobilon; Millipore). Antibodies used were mouse mAb against FANCD2 (1:500, FI17, Santa Cruz), mouse mAb against vinculin (1:5,000, H-10, Santa Cruz), mouse mAb against RAD51 (1:1,000, clone 14B4; Abcam) and rabbit pAb against RAD51 (1:5,000, Professor R. Kanaar, Erasmus MC, Rotterdam), mouse mAb against tubulin (1:5,000, clone B-5-1-2; Santa Cruz), and rabbit pAb against p300 (1:2,500, C-20; Santa Cruz). Secondary antibodies against mouse (1:5,000, Dako, Denmark) and against rabbit (1:5,000, Dako) were used. Precision Plus protein dual-colour standard (161-0374, Bio-Rad) was used as marker for western blots. Protein levels were determined with Image Lab software (v3.01, Bio-Rad Laboratories).

### Protein expression and purification

The expression construct for untagged RAD51^A293T^ was generated by the QuikChange site-directed mutagenesis method by using the wild-type *RAD51* pET11d as template^16^. The construct was sequenced verified. Protein expression and purification were performed using 12-l batches of Rosetta2/pLysS cells containing either expression construct grown at 37 °C until an OD 600 nm reached 0.5. Cells were incubated for another 4 h in the presence of 0.5 mM isopropyl-β-D-thiogalactoside. Purifications steps included (NH_4_)_2_SO_4_ precipitation and chromatography over heparin sepharose and MonoQ columns. Final fractions of the purified proteins were stored at −80 °C in 25 mM Tris-HCl (pH 7.5), 0.5 mM EDTA, 5 mM β-mercaptoethanol, 500 mM NaCl and 5% glycerol^16^.

### D-loop formation assay

Reactions were performed in a final volume of 10 μl containing 5′-end-labelled (Alexa Fluor 488) 90-nt ssDNA (SK3 oligonucleotide^48^, 5′-AATTCTCATTTTACTTACCGGACGCTATTAGCAGTGGCAGATTGTACTGAGAGTGCACCATATGCGGTGTGAAATACCGCACAGATGCGT-3′) at 3.6 μM (nucleotides), 1.2 μM RAD51 (wild type or A293T mutant), 50 mM Tris-HCl (pH 7.5), 1 mM dithiothreitol (DTT), 0.1 mg ml^−1^ acetylated BSA, 30 mM KCl, 2 mM CaCl_2_ and 1 mM ATP^49^. After 5 min incubation at 37 °C, 2 μl of supercoiled pUC19 plasmid DNA at 0.8 mg ml^−1^ (prepared by detergent lysis and purified by double CsCl density-gradient centrifugation) was added and further incubated for 20 min at 37 °C. Reactions were stopped by addition of SDS (1%), EDTA (25 mM) and deproteinized by incubation with proteinase K (1 mg ml^−1^) for 10 min at 37 °C. Reaction mixtures were resolved by 0.7% agarose gel electrophoresis in 0.5 × TB buffer. Gels were analysed using a Typhoon Trio scanner exciting the dye-coupled DNA with a 488-nm laser and detecting emission intensity with a 520-nm BP40 filter at 600 V PMT, 3 mm focal plane. Images obtained were analysed with ImageQuant version 5.2 (Molecular Dynamics).

### ATPase assay

ATPase assays were performed using 1 μM RAD51 or RAD51^A293T^ with or without 30 ng μl^−1^ ϕ × 174 ssDNA. All samples contained 50 mM Tris-HCl (pH 7.5), 1 mM DTT, 200 mM NaCl, 100 ng μl^−1^ acetylated BSA, 2 mM MgCl_2_, 0.2 mM ATP (containing 1 μCi of [γ-32P] ATP) in a final volume of 20 μl. Samples were incubated at 37 °C, and aliquots of 2 μl were taken out at each time point. The reaction was stopped by adding 1 μl of 0.5 M EDTA (pH 8.0) and kept on ice. One microlitre of each sample was spotted on a TLC plate and run in 0.8 M LiCl and 1 M formic acid. Plates were dried and exposed to phosphor imager plates and after 2 h scanned on the Typhoon.

### DNA-binding assay

DNA-binding affinity of wild-type RAD51 and RAD51^A293T^ was compared using ESMA experiments. The assays were performed using 5 nM fluorescently labelled (Alexa Fluor 532) DNA (ss-66nt-AF532 or ds-66 bp-AF532) and a concentration range of wild-type and/or mutant RAD51 up to 4 μM. All samples contained 50 mM Tris-HCl (pH 7.5), 100 mM NaCl, 2 mM CaCl_2_, 1 mM ATP and 1 mM DTT and were incubated for 5 min at 37 °C. Glycerol was added to a final concentration of 4% before loading. Samples were run on a 0.8% agarose gel in 1 × TB (dsDNA) or 5% native PAGE (ssDNA) in 0.5 × TB. Gels were scanned on the Typhoon.

### Scanning force microscopy

The DNA used in the SFM experiments was made by linearizing the 3,901-bp plasmid pDR5 with XmnI^50^. RAD51–DNA complexes were formed in 20-μl reactions containing 3.75 μM DNA (concentration in bp), 1.25 μM RAD51 (wild type or the A293T mutant), 50 mM Tris-HCl (pH 7.5), 2 mM CaCl_2_, 1 mM ATP, 1 mM DTT and 30 or 100 mM KCl as indicated. Reaction mixtures were incubated at 37 °C for 10 min, diluted 10-fold in deposition buffer (10 mM HEPES-KOH (pH 7.5) and 10 mM MgCl2) and deposited on freshly cleaved mica. After 10 s, the mica was washed with water and dried in a stream of filtered air. Images were obtained on a NanoScope IIIa (Digital Instruments; Santa Barbara, CA) operating in tapping mode in air with a type-E scanner. The analysis of nucleoprotein filaments was done using SFMetrics^51^. The contours of filaments were traced manually.

## Additional information

**Accession codes:** The mutation has been deposited to the LOVD database, individual ID 00016307 (http://www.lovd.nl/3.0/home).

**How to cite this article:** Ameziane, N. *et al.* A novel Fanconi anaemia subtype associated with a dominant-negative mutation in *RAD51*. *Nat. Commun.* 6:8829 doi: 10.1038/ncomms9829 (2015).

## Supplementary Material

Supplementary Information Supplementary Figures 1-6, Supplementary Tables 1-6, Supplementary Methods and Supplementary Reference
