# Supplementary material for: A novel Fanconi anaemia subtype associated with a dominant-negative mutation in RAD51
Source: Nat Commun. 2015 Dec 18;6:8829. doi: 10.1038/ncomms9829 (PMC4703882; doi:10.1038/ncomms9829)
Supplement: Supplementary Information — Supplementary Figures 1-6, Supplementary Tables 1-6, Supplementary Methods and Supplementary Reference [file ncomms9829-s1.pdf]

Supplementary Figures

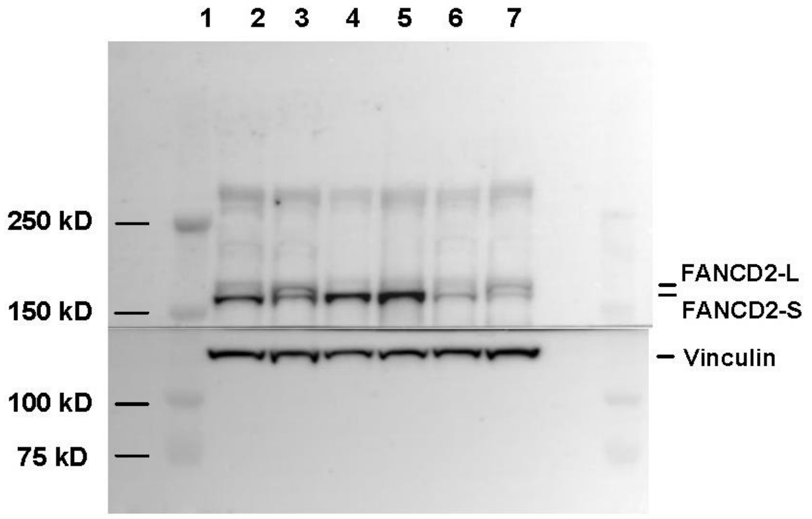

Supplementary Figure 1. FANCD2 Western blot. Lane 1 marker, lane 2 LN9SV, lane 3 LN9SV treated with hydroxyurea (HU), lane 4 GM6914, lane 5 GM6914 treated with HU, lane 6 VU697L, lane 7 VU697L treated with HU. Vinculin was used as the loading control (lower panel). Note that upon treatment with hydroxyurea, in the wild type cell line LN9SV and in the VU697L cell line FANCD2 monoubiquitination (FANCD2-L increased compared to FANCD2-S) was induced. This effect was not observed in the FANCA-deficient cell line GM6914.

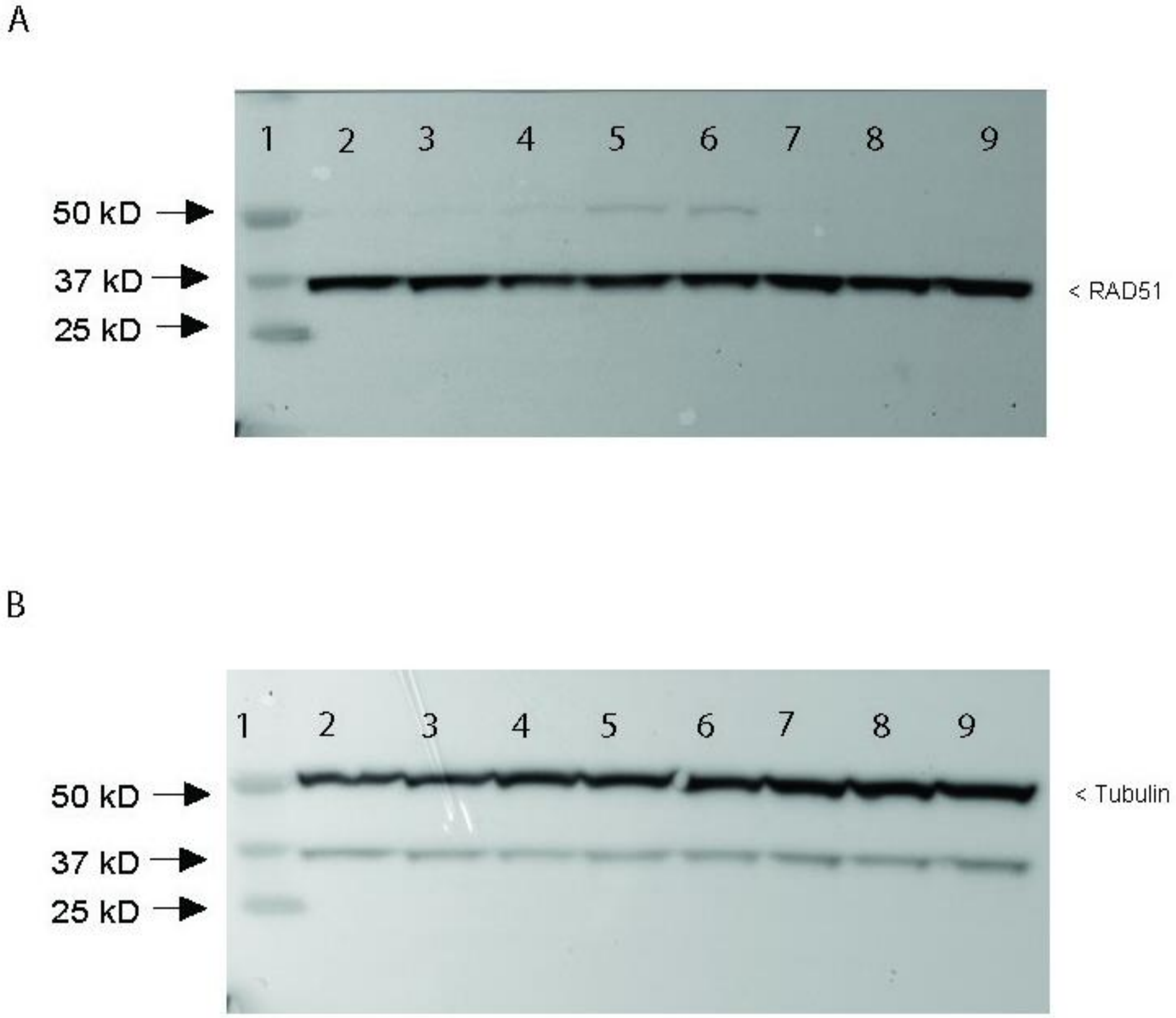

Supplementary Figure 2. <u>RAD51 Western blot</u>. a) Western blot with antibodies against RAD51 analysing lymphoblastoid and fibroblastoid cells lines from the patient (uncropped version of Figure 2d from main text, upper panel). Lane 1 marker, lane 2 HSC93 (WT), lane 3 VU697L, lanes 7 and 8 wild type SV40-immortalized fibroblasts, lane 9 VU697F_SV40, lanes 4-6, not relevant current study. b) Western blot with anti Tubulin antibodies, corresponding to Figure 2d lower panel from main text; further see (a).

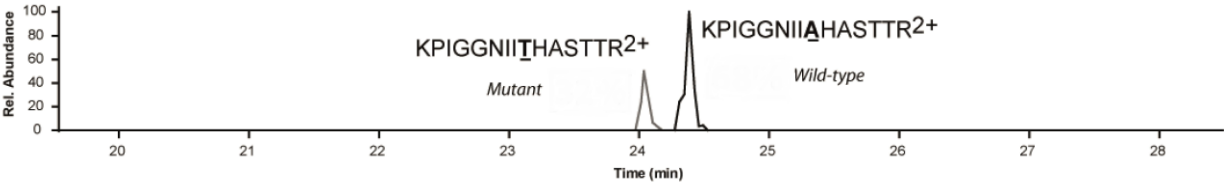

Supplementary Figure 3. Mass spectrometry. Immunoprecipitation of the RAD51 protein was performed on extracts from the patient-derived immortalized fibroblast cell line and control cells, followed by tandem mass spectroscopy analysis. RAD51 was detectable with high sequence coverage (~50%) in both cell extracts. In patient cells both wild type and p.Ala293Thr mutant RAD51 were identified. The relative expression ratio of the two isoforms was assessed by generating extracted ion chromatograms (XIC) of the characteristic KPIGGNII(A/T)HASTTR peptide, which distinguished wild type from mutant RAD51, and calculation of the ratio of XIC peak areas. The p.Ala293Thr mutant comprises 32% of the total protein level in patient cells.

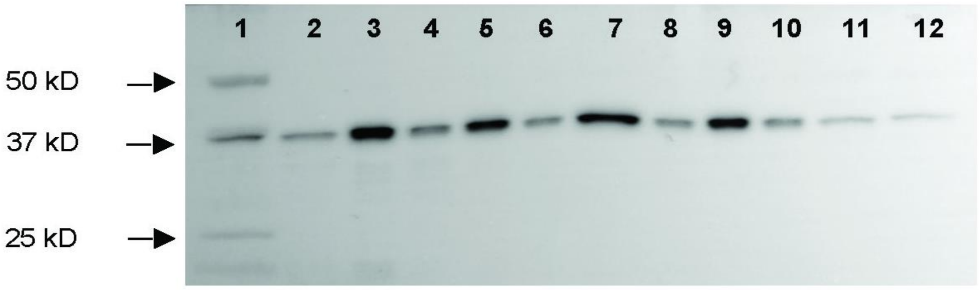

Supplementary Figure 4. RAD51 Western blot. RAD51 Western blot with antibodies against RAD51 analysing inducible cell model (uncropped version of Figure 3e from main text). Lane 1 marker together with untransduced LN9SV wild type cells, lane 2, untransduced LN9SV wild type cells grown in the presence of Mitomycin C (MMC), lanes 3-6 inducible expression of mutant in the absence and presence of MMC, lanes 7-10 inducible expression of wild type RAD51 in the absence and presence of MMC, lanes 11 and 12 GM6914 cells (FANCA mutation) grown in the absence or presence of MMC.

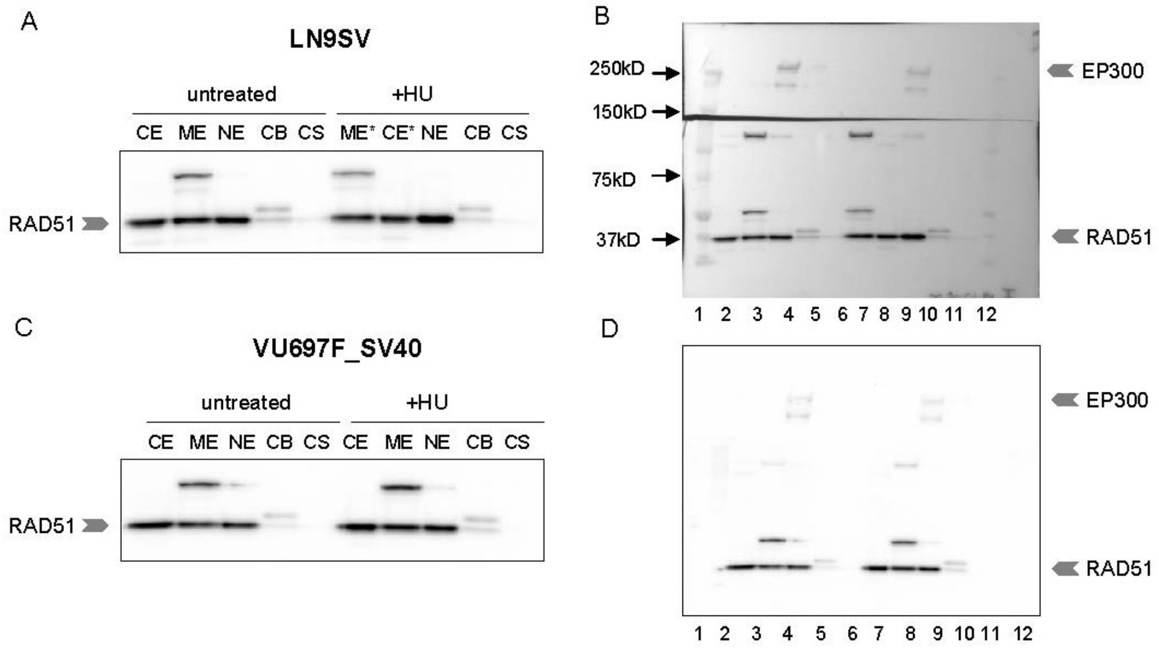

Supplementary Figure 5. Subcellular localization of RAD51. A) Analysis of cytoplasmic (CE), membrane (ME), soluble nuclear (NE), chromatin-bound nuclear (CB), and cytoskeletal (CS) fractions from SV40-transformed wild type fibroblast cells (LN9SV) using antibodies against RAD51, with and without treatment with hydroxyurea (HU) for 24 hours. (Note in the right panel [+HU] the order of loading is different [ME first, followed by CE]). B) Uncropped Western blot of LN9SV analysis. Lane 1 marker, lanes 2-11 (see [a]), lane 12 marker. The upper panel represent the data for EP300 (nuclear control). C) Same analysis as in (A) for the patient-derived cell line VU697F-SV40. D) Uncropped Western blot of VU697F_SV40 analysis (further see (B).

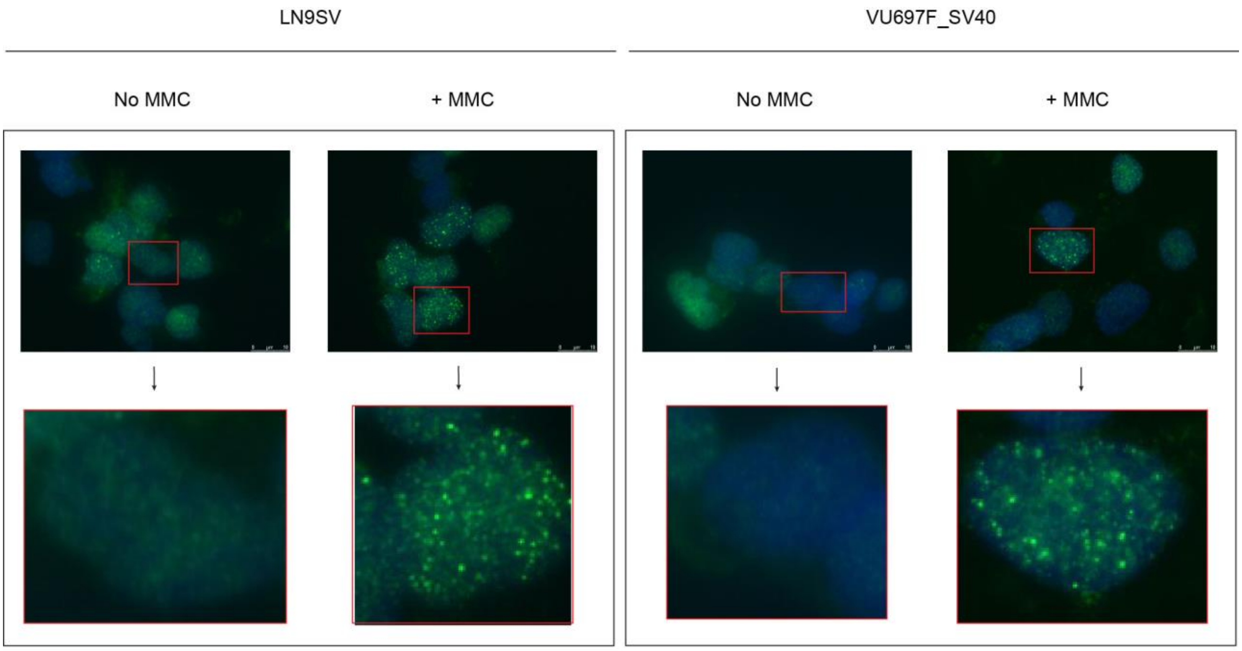

Supplementary Figure 6. Foci formation. RAD51 foci formation observed after incubation in the absence (No MMC) or presence of MMC (+ MMC). Digital zoom-ins of insets are shown in lower panel.

Supplementary Methods

RAD51 foci formation. About 100,000 fibroblast cells were seeded in four-well chamber slides (Nunc)[1]. Cells were grown overnight, washed with PBS, and pre-permeabilized with 0.25% Triton X-100 in PBS. Cells were fixed with 4% paraformaldehyde for 15 at room temperature (RT) and permeabilized with 0.5% Triton X-100 in PBS for 15 min at RT. Cells were treated with PBS + 10% FBS for 1h at RT to block unspecific binding. Slides were incubated with RAD51 pAb (1:2500; Prof. R. Kanaar, Erasmus MC, Rotterdam) in blocking buffer for 2h at RT. Slides were washed with PBS + 0.2% Triton X-100 and incubated with secondary antibody labeled with Alexa 594 (Invitrogen, 1:1000) and DAPI (1:1000) for 1h at RT. The slides were inspected with a fluorescent microscope (DM5000, Leica).

Proteomics. Whole cell extracts were prepared in lysisbuffer (50 mM Tris-HCL, pH7.5, 250 mM NaCl, 5 mM EDTA, 0.1% Triton X-100, supplemented with protease inhibitors). Protein concentration was determined with Protein Standard II and BioRad Protein Assay Dye Reagent Concentrate (Bio-Rad). The beads used for RAD51 immunoprecipitation from 2.5 mg cell lysate protein were reconstituted in XT sample buffer (Bio-Rad) and heated at 95 °C for 7 min. Eluates from the beads were subsequently cleared and concentrated into a small protein band via SDS-PAGE separation. The gel was stained with GelCode Blue stain reagent, followed by excision of the gel bands, reduction of the proteins with DTT and alkylation with iodoacetamide. Proteins were digested with sequencing grade trypsin (3 ng/µL) overnight at 37 °C. Peptides were extracted with acetonitrile, dried in a speed vacuum centrifuge and reconstituted in 10% formic acid prior to mass spectrometry analysis.

Peptides were separated using the Proxeon nLC 1000 system (Thermo Scientific) fitted with a trapping (ReproSil-Pur 120 C18-AQ 3µm [Dr. Maisch GmbH], 100 µm x 200 mm) and an analytical column (ReproSil-Pur 120 C18-AQ 2.4 µm (Dr. Maisch GmbH), 50 µm x 500 mm); both packed in-house. The outlet of the analytical column was coupled directly to a Thermo Orbitrap Fusion (Q-OT-qIT, Thermo Scientific) using the Proxeon nanoflex source. Nanospray was achieved using a distally coated fused silica tip emitter (generated in-house, o.d. 375 µm, i.d. 20 µm) operated at 2.0 kV. Solvent A was 0.1% formic acid/water and solvent B was 0.1% formic acid/acetonitrile. Samples (1

µg) were eluted from the analytical column at a constant flow of 100 nl/min in a 35-min gradient, containing a 16-min linear increase from 7% to 25% solvent B, followed by a 18-min wash at 80% solvent B. Survey scans of peptide precursors from m/z 375-1500 were performed at 120K resolution with a 4 x 105 ion count target. Tandem MS was performed by quadrupole isolation at 1.6 Th, followed by HCD fragmentation with normalized collision energy of 33 and ion trap MS2 fragment detection. The MS2 ion count target was set to 104 and the max injection time was set to 35 ms. Only precursors with charge state 2-6 were sampled for MS2. Monoisotopic precursor selection was turned on; the dynamic exclusion duration was set to 30s with a 10 ppm tolerance around the selected precursor and its isotopes. The instrument was run in top speed mode with 3 s cycles.

Raw data files were processed using Proteome Discoverer (version 1.4.1.14, Thermo Fisher Scientific). MS2 spectra were searched against a custom database (342 entries) containing the Ala293Thr mutant form of RAD51 in addition to the native protein, using Mascot (version 2.4.1, Matrix Science) and Homo sapiens as taxonomy filter. Carbamidomethylation of cysteines was set as fixed modification and oxidation of methionine was set as a variable modification. Trypsin was specified as enzyme and up to two miscleavages were allowed. Data filtering was performed using percolator, resulting in 1% false discovery rate (FDR). Additional filters were search engine rank 1 peptides and ion score >20. To assess the relative composition of wild-type and Ala293Thr mutant RAD51 in the patient-derived cell line versus control cell line, extracted ion chromatograms of the specific distinguishing peptide KPIGGNII(A/T)HASTTR (m/z 768.43 / m/z 783.43) were generated in Thermo Xcalibur Qual Browser software (version 3.0.63).

Supplementary Reference

1. Stoepker, C. et al. SLX4, a coordinator of structure-specific endonucleases, is mutated in a new Fanconi anemia subtype. *Nat. Genet.* **43**, 138-41 (2011).
